# Psychological Experiences of Pregnancy Following Transplantation: A Systematic Qualitative Review

**DOI:** 10.3390/medicina62061072

**Published:** 2026-06-01

**Authors:** Kyriaki-Barbara Papalois, Ermioni Tsarna, Panagiotis Vakas, Sofoklis Stavros, Alkis Matsas, Panagiotis Christopoulos

**Affiliations:** 1 Oxford University Hospitals NHS Foundation Trust, Churchill Hospital Old Rd, Headington, Oxford OX3 7LE, UK; kyriaki.papalois@stx.ox.ac.uk; 2 Second Department of Obstetrics and Gynecology, Aretaieion Hospital, Faculty of Medicine, National and Kapodistrian University of Athens, 11528 Athens, Greece; ermina1990@windowslive.com (E.T.);; 3 Third Department of Obstetrics and Gynecology, General University Hospital Attikon, Faculty of Medicine, National and Kapodistrian University of Athens, 12462 Athens, Greece

**Keywords:** transplantation, pregnancy, psychology, quality of life

## Abstract

*Background and Objectives*: To examine the psychological parameters among pregnant organ transplant recipients that are understudied compared to the physical health of women during post-transplantation pregnancy. *Materials and Methods*: Systematic review based on PubMed, EMBASE, CINAHL, and PsycInfo that were searched until 15 January 2025. Quality Assessment and meta-aggregation were applied to qualitative studies. *Results*: Out of 4361 screened unique studies, six are included. Most studies were retrospective and focused on liver, kidney, and heart transplants. Meta-aggregation identified four synthesized findings: “Perception of Pregnancy after Transplantation”, “Concerns about Maternal Physical Health”, “Concerns about Fetal Health”, and “Emotional Burden by Expectant Mothers and Coping Strategies”. The review was constrained by the potential exclusion of relevant studies due to language restrictions and uncontrolled bias in the included studies. *Conclusions*: Several psychological themes were identified, not all exclusive to transplant recipients. Developing a targeted questionnaire to gather primary data could enhance clinical practice and improve counseling services for this patient population.

## 1. Introduction

Organ transplantation is a transformative procedure for individuals with organ failure, significantly reducing morbidity and mortality while improving overall quality of life [1]. Nevertheless, maintaining a transplanted organ requires lifelong immunosuppression and continuous clinical monitoring [2]. For women of reproductive age, pregnancy following transplantation has been considered feasible since the first documented case in 1958, when Edith Helm conceived and gave birth after receiving a kidney transplantation from her identical twin sister [3]. Subsequent advances in transplantation medicine have enabled many women to successfully conceive and give birth after transplantation. The restoration of reproductive potential after transplantation carries profound personal and psychological implications; however, pregnancy in this context demands careful preconception planning due to the increased maternal and fetal risks. Current guidelines recommend postponing pregnancy for at least one year after transplantation to ensure graft viability and stability. Comprehensive preconception counseling is strongly recommended to enhance health literacy and awareness of pregnancy-related risks among transplant recipients of reproductive age.

Maternal and fetal complications occur more frequently among transplant recipients [4]. The risks for pre-eclampsia, hypertension, diabetes, and graft rejection are all increased [2,5]. Recent evidence indicates that pregnancy does not exert a proven long-term detrimental effect on graft function after kidney transplantation, although transient declines in renal function may occur for up to two years postpartum [6]. Further research is required to determine other potential long-term sequelae. A recent systematic review showed that in pregnancies following solid organ transplantation, the risks for pre-eclampsia, allograft rejection, and allograft loss increase by 5.83-, 2.39-, and 1.55-fold, respectively [7]. In addition, fetal complications such as preterm birth, low birth weight, spontaneous abortion, and stillbirth are reported at higher rates in this population [8]. A systematic review and meta-analysis demonstrated approximately a sixfold increase in the risk for pre-term birth and low birth weight [7]. Concerns have also been raised regarding the potential teratogenicity of certain immunosuppressive agents [8]. Regarding delivery, a cesarean section is more frequently required among transplanted patients [5,9].

Although the clinical implications of pregnancy after organ transplantation are well documented, the psychological burden and patient-reported outcomes remain insufficiently explored. A deeper understanding of the psychological experiences of pregnant transplant recipients is essential to identify the types of support required from experienced professionals. Beyond the common worries associated with pregnancy, transplanted women face a unique constellation of immunological, physiological, and psychological stressors as they strive to maintain both pregnancy and graft integrity. Graft failure secondary to pregnancy-related stress could, hypothetically, lead to organ failure and the need for replacement therapy or further intervention. Enhanced psychological support may help these women navigate uncertainty and anxiety in a secure, structured, and supportive environment. Such interventions have the potential to empower patients, mitigate pregnancy-related anxiety across trimesters, and improve quality of life.

This systematic review utilizes a qualitative meta-aggregation approach to synthesize the subjective experiences and psychological well-being of women during pregnancy following organ transplantation. Within this framework, subjective experiences are operationally defined as the lived experiences, personal narratives, and emotional journeys of pregnancy reported directly by transplant recipients. Correspondingly, psychological well-being is defined as the dynamic emotional states, coping mechanisms, and specific anxieties related to maternal and fetal health during gestation. By mapping these lived experiences and identifying recurrent psychological themes, this review aims to uncover specific areas of vulnerability to inform targeted counseling practices and multidisciplinary support frameworks for this high-risk population.

## 2. Materials and Methods

In accordance with our published protocol, this systematic review initially sought to include original, peer-reviewed research articles of any design (including randomized controlled trials, cohort studies, cross-sectional studies, case–control studies, qualitative descriptive studies, and single-participant qualitative designs) examining both quantitative psychological outcomes, quality of life (QoL), and qualitative experiences during pregnancy following organ transplantation. Eligible studies enrolled adult pregnant participants who were transplant recipients of either solid organ or hematopoietic stem cells. The review primarily focused on solid organ transplantation indicated for chronic disease secondary to organ failure. Although organ donors and uterus transplant recipients were included in the initial search strategy-developed as part of a broader literature search— these records were subsequently excluded. However, our systematic search retrieved no quantitative studies utilizing validated QoL scales or standardized psychological instruments. The available literature consisted exclusively of studies capturing qualitative text data. Therefore, as a necessary deviation from the original protocol, the final analysis, instead of a mixed-methods review, was restricted to a qualitative meta-aggregation focusing on subjective experiences, emotional challenges, and perceived well-being. Literature reviews, systematic reviews, meta-analyses, editorials, letters to the editor, animal studies, and non-English articles were excluded.

A comprehensive literature search was conducted in PubMed, EMBASE, CINAHL, and PsycInfo for potentially eligible studies from inception to 10 June 2023 and was updated on 15 January 2025. The updated search was performed with the assistance of the Bodleian Libraries, University of Oxford. Owing to limited institutional access, the PsycInfo update was performed using OVID instead of EBSCO-host. The search algorithm was initially developed in PubMed and incorporated multiple keywords and MeSH terms related to transplantation, pregnancy, psychology, and quality of life (Table 1). A filter was applied to safely exclude animal studies. During the development phase, this strategy was significantly expanded to explicitly include the names of multiple validated psychological scales and quality of life instruments. While this expansion aimed to maximize search sensitivity to ensure no relevant data were overlooked, it inherently decreased the specificity of the initial retrieval, requiring a more extensive manual screening process. The search algorithm was subsequently adapted for the other databases using the Polyglot Search Translator (Supplementary Table S1) [10]. Lastly, the references of all included studies were manually verified for inclusion.

All identified records were imported into Rayyan, and duplicates were removed following manual verification by two independent reviewers (K.B.P. and E.T.) [11]. Titles and abstracts were screened independently by the same reviewers. Studies that clearly did not meet the inclusion or exclusion criteria were removed at this stage. In cases of disagreement, the record was retained for full-text review. Full-text screening of the remaining potentially eligible articles was conducted independently by K.B.P. and E.T.; discrepancies were resolved by discussion with a third reviewer (P.C.), who made the final decision regarding eligibility.

Data extraction was performed independently and systematically by two reviewers (K.B.P. and E.T.) and tabulated to include author and year of publication, study location, study design, type of transplantation, number of transplanted patients, mean age, number of controls (if applicable), time from transplantation to pregnancy, and pregnancy trimester during which psychological assessment was performed. For qualitative studies, potential bias in data collection was addressed by recording the interviewer’s profession and country. Additional extracted variables included the type of outcome (qualitative or quantitative), study aims and objectives, primary outcomes, main findings, and representative participant quotes from qualitative studies (Supplementary Table S2). Discrepancies in the extracted data were resolved through discussion and consensus with a third reviewer (P.C.).

The Joanna Briggs Institute (JBI) tool for qualitative research was utilized to assess the quality of included qualitative studies [12]. Subsequently, a meta-aggregation approach was applied to synthesize findings and themes, following an inductive process within the JBI Evidence Synthesis framework [12,13]. In more detail, qualitative findings were extracted directly from the primary studies. A finding was defined as a verbatim extraction of the authors’ analytical interpretation of the data, accompanied by supporting illustrations in the form of participants’ direct quotes or the investigators’ recorded observations. These extracted findings, managed using Microsoft Excel ((Version 2604 Build 16.0.19929.20172), were subsequently aggregated into categories based on conceptual and contextual similarities. Finally, these categories were synthesized to develop overarching findings that encapsulate the collective evidence. The meta-aggregation process was conducted independently by two reviewers (K.B.P. and E.T.), and the results were finalized following a consensus meeting with all authors.

To ensure transparency and acknowledge the subjective nature of qualitative synthesis, we explicitly addressed our team’s positionality and potential biases. The review team comprises European obstetricians, several of whom possess extensive clinical experience managing high-risk pregnancies. Prior to the synthesis, the team identified potential assumptions, recognizing that our medical training might inherently bias our interpretations toward clinical psychopathology or risk-management models rather than purely subjective, lived patient experiences. To minimize this interpretive bias, independent coding was performed by two investigators, and disagreements were resolved by mapping findings directly back to the primary authors’ verbatim interpretations and participant illustrations, preventing the imposition of external clinical frameworks. Lastly, debriefing sessions were held to challenge purely clinical assumptions and refocus on the qualitative text data.

Risk of bias assessment in qualitative studies was further evaluated using ConQual scoring, calculated by two independent reviewers (K.B.P. and E.T.), with the level of confidence adjusted according to the dependability and credibility of the findings [13,14]. This systematic review reporting followed the PRISMA guidelines (Supplementary Table S3) [15] and was mapped against the ENTREQ (enhancing transparency in reporting the synthesis of qualitative research) checklist (Supplementary Table S4). This review was registered on PROSPERO on 15 September 2023 (PROSPERO ID CRD42023442475).

## 3. Results

**Figure 1:**
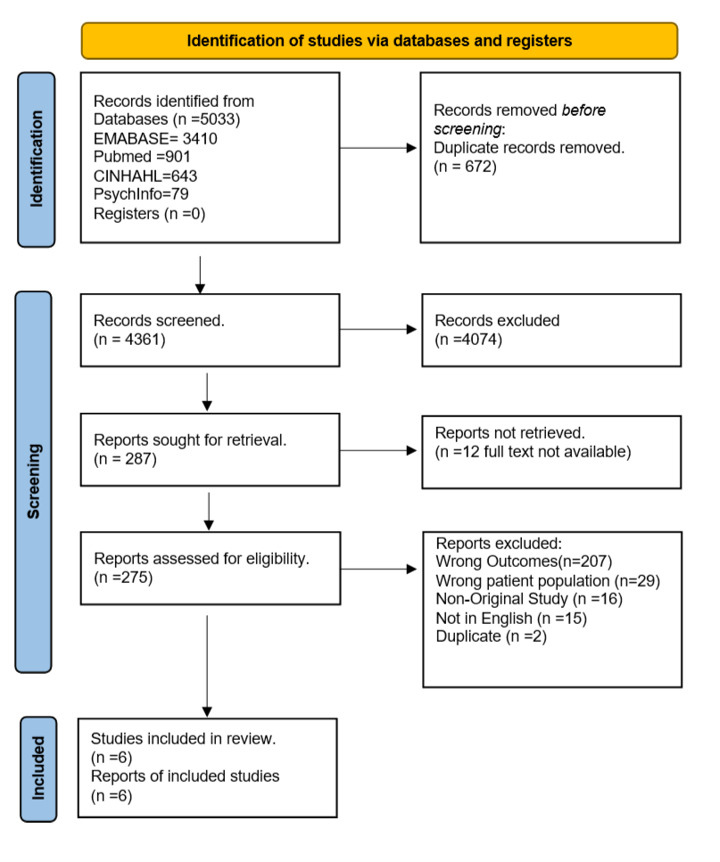
PRISMA Flow diagram showing the number of titles and abstracts identified and screened, and full-text research papers assessed for eligibility and included in the qualitative synthesis.

A total of 5033 records were identified through the systematic search. After removal of 672 duplicates, 4361 titles and abstracts were screened (Figure 1). Of these, 275 records underwent full-text review, resulting in the inclusion of six studies, all of which were qualitative [16,17,18,19,20,21].

**Figure 2:**
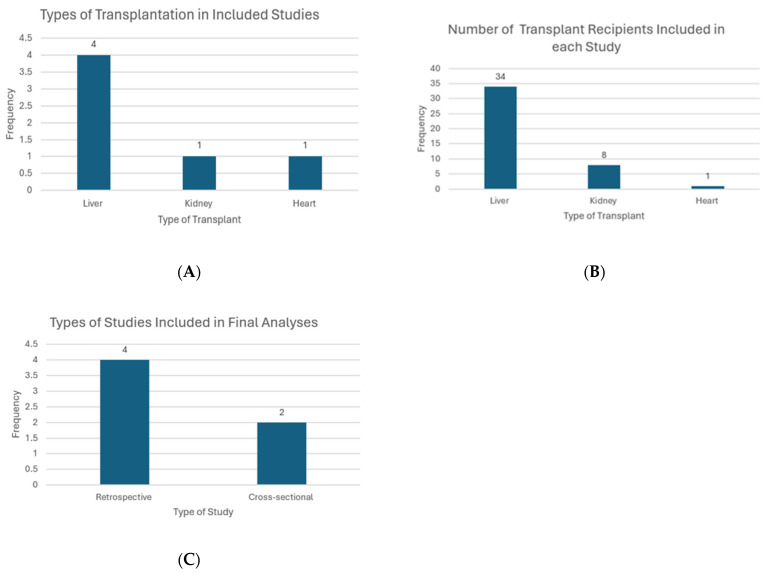
Characteristics of reviewed studies: (**A**) Type of transplantation; (**B**) Number of transplants and recipients in included studies; (**C**) Types of studies included in the final analysis.

All included studies employed a phenomenological theoretical framework, with the majority utilizing semi-structured interviews to identify themes relevant to the outcomes of interest [16,17,18,19,20,21]. Geographically, three studies were conducted in Europe, two in Asia, and one in America, covering the period from 1996 to 2022. Liver transplantation was the most frequently studied transplantation type (four studies), followed by kidney transplantation (one study) and heart transplantation (one study) (Figure 2A). Across all studies, 34 participants had undergone liver transplantation, eight had undergone kidney transplantation, and one had undergone heart transplantation (Figure 2B). The predominant study design was retrospective (four studies), followed by cross-sectional (two studies) (Figure 2C).

The JBI Quality Critical Appraisal Checklist for Qualitative Research was applied to all included qualitative studies (Supplementary Table S5). Areas most frequently requiring further clarification included “congruity between philosophical perspective and research methodology” and “locating the researcher culturally and theoretically”. Findings from qualitative studies were supplemented with direct participant quotes. Similar findings were grouped into categories, which were further synthesized into broader themes, termed as synthesized findings (Supplementary Tables S6 and S7).

In total, nine categories were identified, namely: (i) Perception of Pregnancy by others (family, friends, people with similar experiences), (ii) Expectant Mothers’ Perception of their Pregnancy, (iii) Fear of Complications, Damage, or Failure of the Graft, (iv) Anxiety about Birth Plan and Childbirth, (v) Physical Health Status of Pregnant Women, (vi) Fear and Anxiety Related to Fetal Loss, Malformations, and Genetic Disease, (vii) Concerns about Fetal Immunosuppression, (viii) Emotional Burden of Pregnancy on Expectant Mothers, and (ix) Coping Strategies for Anxiety.

From the aforementioned nine categories, four synthesized findings were generated, namely: (i) Perception of Pregnancy, (ii) Concerns about Maternal Physical Health, (iii) Concerns about Fetal Health, and (iv) Emotional Burden by Expectant Mothers and Coping Strategies. These synthesized findings, their underlying categories, and the supporting findings are presented in detail below. Findings are expressed as either direct participants’ quotes or authors’ conclusions drawn from participant interviews, denoted as authors’ conclusions (AC).

### 3.1. Perception of Pregnancy After Transplantation

**Figure 3:**
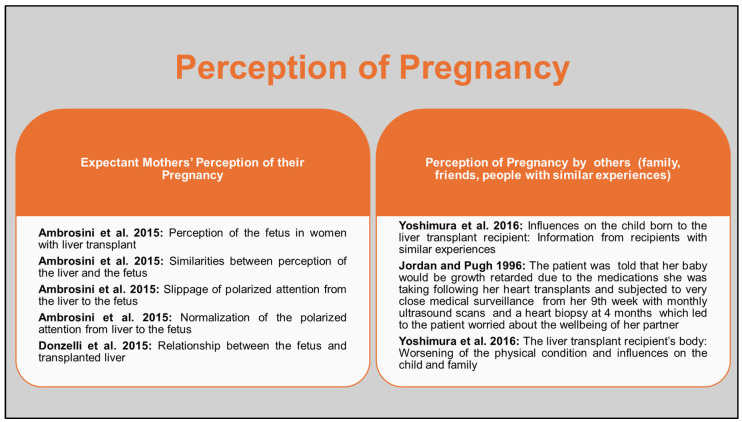
Perception of Pregnancy: Contributing findings and categories [16,17,18,21].

The synthesized finding “Perception of Pregnancy after Transplantation” encompassed the categories “Expectant Mothers’ Perception of their Pregnancy” [16,17] and “Perception of Pregnancy by others (family, friends, and people with similar experiences)” [16,18,21] (Figure 3).

#### 3.1.1. Expectant Mothers’ Perception of Their Pregnancy

Pregnant women described similar perceptions of the fetus and the transplanted organ, reflecting concern about the effect of pregnancy on the integrity of their graft and potential associated risks [16]. Another liver transplant recipient remarked that they were intensely aware of their child, highlighting a heightened awareness of pregnancy following transplantation [16].

Studies indicated that prior to pregnancy, women’s primary focus was on their transplanted organ; however, this attention shifted toward the fetus once pregnancy began [16]. Likewise, another study confirmed that while participants remained aware of their transplanted organ, maternal vigilance transitioned predominantly toward fetal health [17].

Women also expressed profound gratitude for their transplant, which enabled them to conceive [16].

#### 3.1.2. Perception of Pregnancy by Others (Family, Friends, and People with Similar Experiences)

Transplanted pregnant women described a desire for information from other women with similar medical histories and experiences and emphasized the psychological impact of their pregnancy on their families, indicating a wish to understand the long-term development of children born to parents with a similar medical problem [21]. Additionally, concerns about the well-being of their partners in the event of maternal mortality were noted [21]. These anxieties were compounded by worries related to the recipients’ complex medical histories [21]. One participant vividly described her partner’s perception of her pregnancy and fear of loss of their partner due to the high-risk of pregnancy [18].

### 3.2. Concerns About Maternal Physical Health

**Figure 4:**
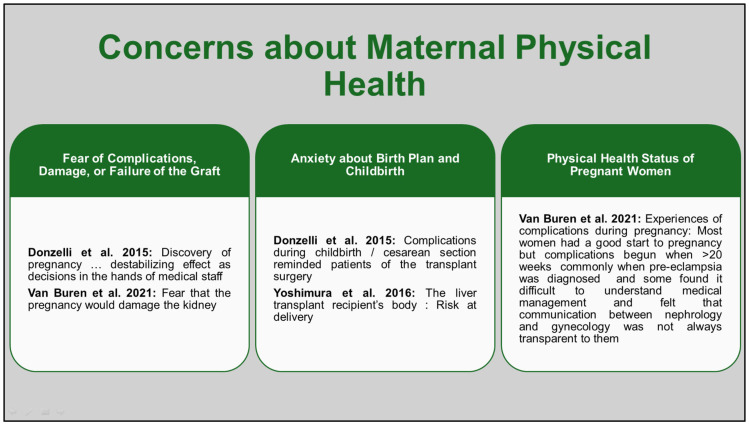
Concerns about Maternal Physical Health: Contributing findings and categories [17,20,21].

The synthesized finding “Concerns about Maternal Physical Health” encompassed the categories “Fear of Complications, Damage, or Failure of the Graft” [17,20], “Anxiety about Birth Plan and Childbirth” [17,21], and “Physical Health Status of Pregnant Women” [20] (Figure 4).

#### 3.2.1. Fear of Complications, Damage, or Failure of the Graft

Transplanted pregnant women expressed fear of graft failure during pregnancy and described feeling their decision regarding continuation of pregnancy rested primarily with medical professionals rather than themselves [17]. A kidney transplant recipient similarly reported concern for their graft due to the growing fetus [20].

#### 3.2.2. Anxiety About Birth Plan and Childbirth

Anxiety surrounding labor and delivery was frequently reported, often linked to memories of transplantation and fears of medical complications [17]. Among liver transplant recipients, major concerns included tolerability and fear of the birth, as well as bleeding risk and sensitization following the need for potential blood transfusions [21].

#### 3.2.3. Physical Health Status of Pregnant Women

This category captured participants’ concerns regarding post-surgical complications and pregnancy-related health issues, particularly in the second or third trimester. Concerns were expressed regarding pregnancy complications, such as preeclampsia, occurring more frequently among transplant recipients [20].

### 3.3. Concerns About Fetal Health

**Figure 5:**
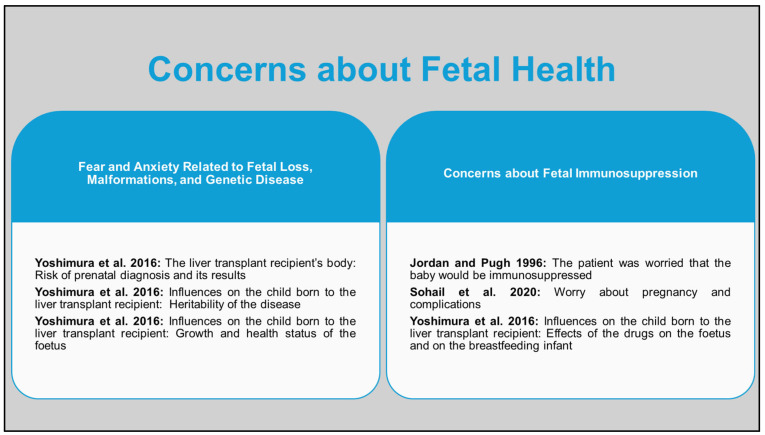
Concerns about Fetal Health: Contributing findings and categories [18,19,21].

The synthesized finding “Concerns about Fetal Health” encompassed the categories “Fear and Anxiety Related to Fetal Loss, Malformations, and Genetic Disease” [21] and “Concerns about Fetal Immunosuppression” [18,19,21] (Figure 5).

#### 3.3.1. Fear and Anxiety Related to Fetal Loss, Malformations, and Genetic Disease

Participants expressed persistent anxiety regarding miscarriage and fetal loss. Frequent monitoring during pregnancy and invasive procedures, such as amniocentesis for genetic testing, were sources of significant worry due to the risk of miscarriage [21]. Concerns about fetal development were also evident [21]. Additionally, persistent fears about the heritability of disease were reported even after medical reassurance [21].

#### 3.3.2. Concerns About Fetal Immunosuppression

Concerns regarding the potential teratogenic or developmental effects of immunosuppressive therapy on the fetus and their potential impact on lactation were common [21]. The uncertainty surrounding immunosuppression was further echoed by Sohail et al., where a participant shared fears of conceiving on immunosuppressive medication, given her complex medical history [19]. Similarly, another participant following cardiac transplantation expressed worry of transmitting an immunosuppressed state to the child and exposing them to a higher risk of infection [18].

### 3.4. Emotional Burden by Expectant Mothers and Coping Strategies

**Figure 6:**
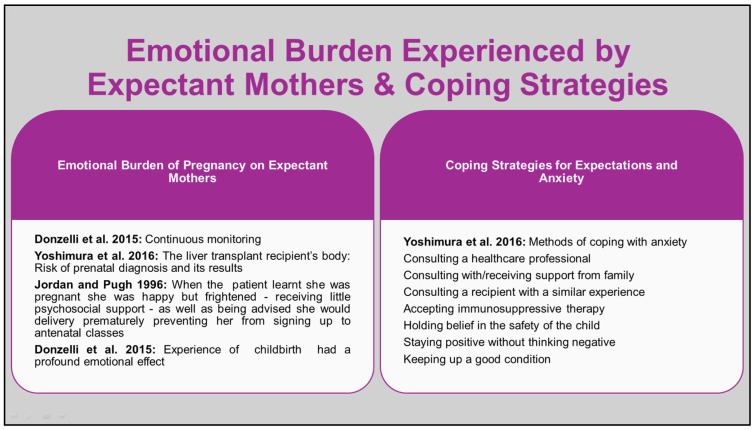
Emotional Burden Experienced by Expectant Mothers and Coping Strategies: Contributing findings and categories [17,18,21].

The synthesized finding “Emotional Burden Experienced by Expectant Mothers and Coping Strategies” derived from the categories “Emotional Burden of Pregnancy on Expectant Mothers” [17,18,21] and “Coping Strategies for Expectations and Anxiety” [21] (Figure 6).

#### 3.4.1. Emotional Burden of Pregnancy on Expectant Mothers

Participants emphasized the psychological strain associated with continuous monitoring, the potential for unfavorable results of invasive prenatal testing, and the eventuality of a medically indicated abortion or preterm birth. Increased surveillance created dilemmas and anxiety, as participants felt the decisions surrounding their pregnancy rested in the hands of healthcare professionals [17]. Related key themes included concerns related to risks associated with amniotic fluid testing, potential miscarriage, and the possibility of termination following any abnormal results [21]. A heart transplant recipient, initially excited, expressed her anxiety about delivering prematurely, which prevented her from attending antenatal classes [18]. Despite these challenges, many transplanted women expressed profound gratitude for their pregnancy and childbirth experiences [17].

#### 3.4.2. Coping Strategies for Expectations and Anxiety

Coping strategies included seeking advice from healthcare professionals, family members, and peers with similar experiences [21]. Participants emphasized the importance of understanding immunosuppression risks and benefits, holding a belief in the child’s safety, maintaining a positive outlook, and keeping up a good condition [21].

Consultations with healthcare professionals, such as doctors, obstetricians, internists, midwives, and nurses, were extremely valuable [21]. Women also relied on family members, particularly their partners and their mother, and other transplant recipients, for emotional support [21].

Furthermore, strategies for managing anxiety included weighing the benefits and risks of continuing immunosuppressive therapy [21]. Transplanted pregnant women reported that to accept the risks of immunosuppression for the fetus, it was important to understand the risks of immunosuppression discontinuation or dose reduction for themselves and their graft and how that would eventually affect the fetus [21].

A further strategy involved fostering a positive mindset during pregnancy and focusing on what patients could control [21]. Maintaining physical well-being through diet and weight management, infection prevention, and self-care was perceived as vital for maternal and fetal health [21].

#### 3.4.3. Quality Assessment of the Included Studies

To evaluate the confidence in the qualitative meta-aggregation synthesis, the ConQual framework was applied. The ConQual scores were low for all synthesized findings (Supplementary Table S8). Initially rated as high, all synthesized findings were downgraded for dependability and credibility. Regarding dependability, most findings were downgraded by one level to Moderate, due to incomplete adherence to five JBI criteria (questions 2, 3, 4, 6, and 7) (Supplementary Table S5). None of the studies addressed the researcher’s influence on the research and vice versa, while most studies did not locate the researcher culturally or theoretically. Occasionally, issues arose regarding the congruity between the research methodology and the representation and analysis of data. In terms of credibility, all findings dropped an additional level to low, as some of the supporting illustrations used to formulate the categories were not direct participant quotes but rather authors’ interpretations and conclusions. Specifically, the study by Yoshimura et al. contributed to several categories and included indirect quotes from participants [21].

## 4. Discussion

This systematic review examined the experiences and psychological burden of pregnancy among transplant recipients. Four synthesized findings were identified from six qualitative studies: Perception of Pregnancy, Concerns about Maternal Physical Health, Concerns about Fetal Health, and Emotional Burden Experienced by Expectant Mothers and Coping Strategies. However, confidence was low for all the aforementioned synthesized findings.

### 4.1. Shared and Unique Psychosocial Dimensions

To optimize clinical management, it is essential to delineate which aspects of the post-transplant pregnancy experience overlap with broader obstetric populations and which are uniquely specific to transplant recipients. Many identified themes, including anxiety regarding miscarriage, childbirth, and fetal malformations, mirror the frequent experiences of nulliparous women, those with prior obstetric trauma, or high-risk pregnancies [22,23]. Similarly, concerns related to maternal physical health are encountered in individuals navigating advanced maternal age with standard medical comorbidities. However, pregnancy-associated risks are heightened among transplant recipients. Liver transplant recipients are more prone to postpartum hemorrhage, while gestational hypertension and pre-eclampsia occur more frequently in kidney transplant recipients [24,25,26,27]. The reliance on evidence-based coping mechanisms, such as consulting healthcare professionals, seeking support from family, friends, and individuals with similar experiences, maintaining positivity and self-efficacy, and sustaining a healthy lifestyle, aligns with general prenatal psychological adaptations [27].

However, transplantation introduces highly distinct psychosocial challenges as well. While general high-risk obstetric patients worry about gestational complications, transplant recipients navigate a profound dual vulnerability: balancing fetal safety against lifelong graft survival. A defining feature of this population is the acute ‘attention shift’ from the transplant to the fetus. Once pregnant, maternal vigilance transitions predominantly toward fetal health, often causing patients to rationalize substantial personal health risks to protect the pregnancy. While a similar emotional trajectory of ‘intransigent guilt’, decisional conflict, and ‘withholding emotional investment’ due to viability fears has been documented in broader chronic kidney disease cohorts, transplant recipients experience an amplified fear of maternal mortality [28,29]. Physiological adaptations of pregnancy, including increased cardiac output, hepatic collateral circulation, and alterations in electrolyte balance and fluid retention, impose additional strains on transplanted organs, particularly the heart, liver, and kidneys. Because solid organ transplantation is a life-saving intervention, the prospect of graft failure directly threatens maternal life expectancy, triggering anxiety over immediate survival, the future welfare of existing children, and the potential impact on the patient’s support network.

Furthermore, medical management introduces a unique pharmacological burden. Physiologically, a successful pregnancy requires immunological adaptation to tolerate the semi-allogeneic fetus [30], while lifelong immunosuppressive therapy remains necessary for transplant recipients to prevent graft rejection and poses potential risks for both maternal and fetal health. These agents can increase susceptibility to infections, organ dysfunction, and pregnancy-related complications [31]. While general high-risk patients may also worry about medication safety, transplant recipients experience severe, specific anxieties regarding the teratogenicity of essential immunosuppressive regimens, fetal growth, and the long-term impact of these agents on breastfeeding and infant development [18,32]. Indeed, increased rates of fetal complications, including fetal mortality, prematurity, and growth restriction, have been reported in liver transplant recipients [25]. Therefore, pregnancy in transplant recipients must be carefully planned to minimize fetal exposure to teratogenic agents such as mycophenolate [32]. It should be noted that the intensive clinical surveillance required to monitor immunosuppressive medications and organ function during pregnancy acts as a paradox; it provides reassurance of fetal safety while simultaneously provoking anxiety by constantly reinforcing the patient’s medical vulnerability.

### 4.2. Theoretical and Phenomenological Interpretations of Post-Transplant Pregnancy Experience

A deeper insight into the internal conflicts and complex meanings transplant recipients attribute to pregnancy may be gained by evaluating these lived experiences through phenomenology and established psychological frameworks. In the context of Illness Identity Theory, the maternal attention shift from the graft to the fetus suggests elements of acceptance and enrichment, indicating that successful adjustment to post-transplant life and renewed physical health enabled the desire for pregnancy [16,33]. However, a profound sense of interdependence emerges, as women parallel the vulnerability of the graft with that of the fetus [17]. This delicate balance frequently induces ambivalence, defined by the simultaneous gratification of pregnancy coupled with an intense fear of harming either the graft or the fetus [34]. Consequently, the introduction of pregnancy can trigger a biographical disruption, where the sustained period of good health that originally permitted conception is suddenly perceived as a paradox that endangers the graft and overall maternal survival [21,35].

This psychological burden is heavily compounded by the medicalization and intensive surveillance of high-risk obstetric care [36]. While meant to reassure, frequent monitoring creates a complex interplay between dependency and autonomy, occasionally exerting a destabilizing effect as critical decisions are perceived to rest entirely with medical staff [17,37,38]. This perceived loss of autonomy, alongside conflicting or unclear information from fragmented multidisciplinary teams, exacerbates “uncertainty in illness” [20,39]. Furthermore, the integration of the graft reflects a deep physical embodiment; the felt responsibility to sustain both the life-saving graft and the developing fetus increases maternal pressure [20,40]. This vulnerability is further magnified during childbirth preparation, where mandated surgical interventions can inadvertently trigger past trauma from previous transplant-related surgeries [17,21].

Unique ethical and psychosocial dilemmas also center on pharmacological and hereditary anxieties. Expectant mothers experience anxiety regarding the teratogenicity of essential immunosuppressive regimens and the potential long-term impacts of child immunosuppression during breastfeeding [18]. For conditions with an underlying genetic etiology, this is worsened by fears of disease heritability [21]. Navigating these complex reproductive ethics can expose patients to perceived social stigma regarding their reproductive choices, which may indirectly drive “overprotective” parenting or “overparenting” behaviors in the future [34].

When managing these multi-layered stressors, patients utilize distinct problem-focused and emotion-focused coping mechanisms, as outlined by the Transactional Model of Stress and Coping [41]. Problem-focused strategies predominantly involve actively seeking transparent clinical information and consulting peer networks of transplant recipients who share similar pregnancy experiences [21]. Conversely, emotion-focused coping centers on cognitive reframing, maintaining positivity, and holding a firm belief in the safety of the child [21]. Because the compounding weight of anxieties surrounding post-transplant pregnancy may potentially render these coping strategies ineffective or maladaptive, targeted psychological interventions focusing on illness identity, ambivalence, and perceived medical uncertainty could play a valuable role in helping mitigate the risk of perinatal mental health issues.

### 4.3. Clinical Implications and Actionable Strategies

The findings of this review indicate that standard high-risk obstetric care models should be adapted to address the specific vulnerabilities of transplant recipients. First, because unplanned pregnancies carry decisional insecurity, proactive preconception counseling is important. Evidence suggests that pregnancy risks are frequently underestimated by patients with chronic diseases, and pre-pregnancy counseling rates remain critically low [42]. Second, care progression beyond standard multidisciplinary medical monitoring to integrate dedicated psychological and genetic counseling, which may be beneficial. This support should not be prescriptive; rather, communication regarding medical risks must be balanced with respect for patient autonomy and personal values. To this end, the incorporation of validated tools such as the Mother’s Autonomy in Decision Making (MADM) and the Norwegian Pregnancy and Maternity Care Patients’ Experiences Questionnaire (PreMaPEQ) may prove helpful [43,44]. To reconcile the differential risk perceptions between the clinical team and the patient, counselling should be highly personalized, tailoring the depth of information to the individual’s coping capacity to prevent clinical appointments from becoming psychologically overwhelming [28,45]. Finally, the review highlights a critical, unmet practical need, namely access to peer-led support networks and qualitative testimonials from other transplant recipients who have experienced pregnancy [21]. Future research should explore the development of structured peer-support frameworks and investigate whether they can better capture and support patient autonomy in this uniquely high-risk population.

### 4.4. Strengths and Limitations of the Systematic Quality Review

This systematic qualitative review aimed to explore psychological well-being and quality of life during pregnancy among transplant recipients. The protocol for this systematic review was prospectively registered on PROSPERO, and all amendments were published, ensuring methodological transparency. Strengths of this systematic review include an extensive, systematic, and comprehensive search strategy, the rigorous risk of bias assessment of the reviewed studies, and the formal quality appraisal of the synthesized findings.

Despite these strengths, several limitations must be acknowledged. Methodologically, the search strategy did not utilize proximity operators, and the formal searches of gray literature or manual journal hand-searching were not performed. However, to ensure comprehensive coverage within the searched databases, the keyword algorithm itself was developed to be highly expansive, incorporating a wide array of specific psychological and quality-of-life terms. While this broad approach maximized search sensitivity to capture the potentially relevant literature, it inherently reduced specificity, resulting in a large initial pool of 4361 unique records that ultimately yielded just six eligible studies. The overwhelming majority of these records focused on outcomes that were not relevant for our review (e.g., biomedical, clinical, pharmacological), and the final yield of just six eligible studies fundamentally highlights a pronounced scarcity of research exploring the subjective experiences of pregnant transplant recipients. Furthermore, the application of a language criterion may have resulted in the exclusion of relevant studies published in languages other than English, potentially introducing language bias, and full-text unavailability precluded the inclusion of some records. While the majority of international research is published in English, high-quality data are also published in other languages, including Spanish, Chinese, Portuguese, and French. This is particularly relevant for our systematic review, as it may have affected the generalizability of our conclusions in culturally diverse settings.

The substantial clinical and contextual heterogeneity inherent across the included primary studies must be carefully considered when interpreting the findings of our systematic qualitative review. The final data pool synthesizes experiences across diverse solid organ transplant recipients, predominantly through liver, who navigated varying immunosuppressive regimens and clinical protocols. Beyond these medical variables, the studies span across geographical regions, reflecting fundamentally different healthcare delivery models, socioeconomic support systems, and cultural paradigms surrounding both transplantation and maternity. Additionally, even though the majority of pregnancies occurred in the last 15 years, the oldest data originated 30 years ago. Although these multi-layered variations do not invalidate the synthesized findings, they do introduce complex contextual variations that limit the immediate generalizability of our conclusions to all transplant populations universally.

Due to the absence of quantitative studies, meta-analysis and subgroup analyses were not feasible. Furthermore, analysis by transplanted organs could not be performed, as most available data pertained to liver transplant recipients, in contrast to our expectation that kidney transplant recipients would be represented in the majority of studies, given the higher prevalence of kidney transplants. Most included studies were retrospective, introducing potential recall bias and precluding analysis by pregnancy trimester. The average time from transplantation to pregnancy and the pregnancy trimester at the time of data collection were not reported in most studies, limiting stratified analysis of the findings. Moreover, the cultural and theoretical positioning of the interviewers was not disclosed in the reviewed studies, precluding evaluation of interviewer bias. Notably, no eligible studies involved bone marrow transplant recipients; thus, the findings of this review apply exclusively to solid organ transplant recipients. Finally, some qualitative findings relied on credible but non-unequivocal illustrations, as it was unclear whether the reported conclusions were direct participant quotes or author interpretations.

## 5. Conclusions

The psychological well-being of women during pregnancy following organ transplantation appears to be shaped by their individual perception of pregnancy and varying concerns regarding maternal and fetal health. In the reviewed literature, women frequently expressed anxiety regarding fetal growth, the potential teratogenic and immunological effects of immunosuppressive therapy, and risks associated with prematurity. Maternal health concerns primarily revolved around the body’s perceived capacity to sustain pregnancy, potential impacts on graft function, and labor-associated complications. While frequent monitoring and close medical surveillance are standard clinical practice, some reports suggest that these interventions may paradoxically intensify anxiety in certain individuals. Despite these challenges, overall satisfaction with the pregnancy experience was often reported, particularly when multidisciplinary support and empathetic communication were in place.

Pre-pregnancy and early pregnancy counseling may help address specific fears through evidence-based guidance and exposure to peer experiences. The implementation of validated instruments, such as the PreMaPEQ and MADM, or the development of transplant-specific assessment tools, could potentially support a more structured evaluation of psychological outcomes and help inform future counseling frameworks. Ultimately, support during pregnancy should ideally be informed by the documented experiences of women in this high-risk obstetric population. While medical expertise is essential for safety, these findings highlight the importance of respecting individual autonomy and values, suggesting that psychological support should be tailored to the specific needs of each woman.

## Figures and Tables

**Table 1:** The search algorithm in PubMed.

(“transplantation”[MeSH Terms] OR “transplant recipient*”[MeSH Terms] OR “transplantation”[Title/Abstract] OR “grafting”[Title/Abstract] OR “graft*”[Title/Abstract]
AND
(“pregnancy”[MeSH Terms] OR “pregnan*”[Title/Abstract] OR “gestation”[Title/Abstract] OR “prenatal”[Title/Abstract] OR “intrauterine”[Title/Abstract] OR “in utero”[Title/Abstract] OR “Perinatal”[Title/Abstract] OR “Postnatal”[Title/Abstract])
AND
(“quality of life”[MeSH Terms] OR “Psychology” [MeSH Terms] OR “behavioral medicine”[MeSH Terms] OR “Psychological Well-Being”[MeSH Terms] OR “Patient Reported Outcome Measures”[MeSH Terms] OR “Interpersonal Relations”[MeSH Terms] OR “Mental Disorders”[MeSH Terms] OR “Adaptation, Psychological”[MeSH Terms] OR “well being”[Title/Abstract] OR “Patient Health Questionnaire”[MeSH Terms] OR “psychometrics*”[MeSH Terms] OR “Patient Outcome Assessment”[MeSH Terms] OR “Mental Health”[MeSH Terms] OR “psychological”[Title/Abstract] OR “psychosocial”[Title/Abstract] OR “emotional”[Title/Abstract] OR “depress*”[Title/Abstract] OR “anxiety”[Title/Abstract] OR “quality of life”[Title/Abstract] OR “quality-of-life”[Title/Abstract] OR “QoL”[Title/Abstract] OR “hql”[Title/Abstract] OR “hqol”[Title/Abstract] OR “h qol”[Title/Abstract] OR “hrqol”[Title/Abstract] OR “hr qol”[Title/Abstract] OR “Patient Reported Outcome Measure*”[Title/Abstract] OR “PROMs”[Title/Abstract] OR “PREMs”[Title/Abstract] OR “Patient Reported Experience Measure*”[Title/Abstract] OR “well-being”[Title/Abstract] OR “wellbeing”[Title/Abstract] OR “Generalised Anxiety Disorder”[Title/Abstract] OR “GAD”[Title/Abstract] OR “GAD-2”[Title/Abstract] OR “GAD-7”[Title/Abstract] OR “Hamilton Anxiety Scale”[Title/Abstract] OR “HAM-A”[Title/Abstract] OR “Hamilton Depression Scale”[Title/Abstract] OR “HAM-D”[Title/Abstract] OR “Patient Health Questionnaire”[Title/Abstract] OR “PHQ”[Title/Abstract] OR “PHQ-2”[Title/Abstract] OR “PHQ-9”[Title/Abstract] OR “Hospital Anxiety and Depression Scale”[Title/Abstract] OR “HADS”[Title/Abstract] OR “HADS-A”[Title/Abstract] OR “HADS-D”[Title/Abstract] OR “Self-Acceptance Scale”[Title/Abstract] OR “SESA”[Title/Abstract] OR “Symptom Checklist-90”[Title/Abstract] OR “SCL-90”[Title/Abstract] OR “sf”[Title/Abstract] OR “shortform”[Title/Abstract] OR “short form”[Title/Abstract] OR “WHOQol-Bref”[Title/Abstract] OR “State-Trait Anxiety Inventory”[Title/Abstract] OR “STAI”[Title/Abstract] OR “Beck Depression Scale”[Title/Abstract] OR “BDI”[Title/Abstract] OR “Beck Anxiety Inventory”[Title/Abstract] OR “BAI”[Title/Abstract] OR “Brief Symptom Inventory”[Title/Abstract] OR “BSI”[Title/Abstract] OR “Rosenberg’s Self-Esteem Questionnaire”[Title/Abstract] OR “RSES”[Title/Abstract] OR “QOLS”[Title/Abstract] OR “McGill Quality of Life Questionnaire”[Title/Abstract] OR “MQOL”[Title/Abstract] OR “MQOL-E”[Title/Abstract] OR “Health Related Quality of Life”[Title/Abstract] OR “Mood Disorder Questionnaire”[Title/Abstract] OR “MDQ”[Title/Abstract] OR “Montgomery-Asberg Depression Scale”[Title/Abstract] OR “MADRS”[Title/Abstract] OR “World Health Organization Quality of Life Instrument”[Title/Abstract] OR “WHOQOL-BREF”[Title/Abstract] OR “Global Quality of Life Scale”[Title/Abstract] OR “nottingham health profile”[Title/Abstract] OR “sickness impact profile”[Title/Abstract] OR “Eq”[Title/Abstract] OR “Euroqol”[Title/Abstract] OR “euro qol”[Title/Abstract] OR “Eq5d”[Title/Abstract] OR “eq 5d”[Title/Abstract])
NOT
(“animals”[MeSH Terms] NOT “humans”[MeSH Terms])

Note: * indicates truncation used to capture multiple word variants sharing the same root.

## Data Availability

The original contributions presented in this study are included in the article/Supplementary Materials. Further inquiries can be directed to the corresponding author.

## Supplementary Materials

The following supporting information can be downloaded at https://www.mdpi.com/article/10.3390/medicina62061072/s1. Table S1: The search algorithm in Embase, CINHAL, and PsycInfo. Table S2: Summary of findings of the reviewed studies. Table S3: PRISMA checklist. Table S4: ENTREQ checklist. Table S5: Quality Assessment of reviewed qualitative studies, based on the Joanna Briggs Institute Critical Appraisal Tool. Table S6: Findings and illustrations contributing to the categories developed during meta-aggregation. Table S7: Synthesized findings, contributing categories, and findings with illustrations. Table S8: ConQual Scores for Each Synthesized Finding.
